# Influence of air exposure on structural isomers of silver nanoparticles

**DOI:** 10.1038/s42004-023-00813-9

**Published:** 2023-01-24

**Authors:** Jerome Vernieres, Nathalie Tarrat, Sean Lethbridge, Erica Watchorn-Rokutan, Thomas Slater, David Loffreda, Richard E. Palmer

**Affiliations:** 1 Nanomaterials Lab, Mechanical Engineering, Swansea University, Bay Campus, Fabian Way, Swansea, SA1 8EN UK; 2 CEMES, Université de Toulouse, CNRS 29 Rue Jeanne Marvig, 31055 Toulouse, France; 3 School of Chemistry, Cardiff University, Main Building, Park Place, Cardiff, CF10 3AT UK; 4 ENSL, CNRS, Laboratoire de Chimie UMR 5182, 46 Allée d’Italie, 69364 Lyon, France; 5 Present Address: Department of Physics, Technical University of Denmark, Kongens Lyngby, Lyngby, Denmark

**Keywords:** Structural properties, Atomistic models, Nanoparticles

## Abstract

Up to date, the influence of ambient air exposure on the energetics and stability of silver clusters has rarely been investigated and compared to clusters in vacuum. Silver clusters up to 3000 atoms in size, on an amorphous carbon film, have been exposed to ambient air and investigated by atomic-resolution imaging in the aberration-corrected Scanning Transmission Electron Microscope. Ordered structures comprise more than half the population, the rest are amorphous. Here, we show that the most common ordered isomer structures is the icosahedron. These results contrast with the published behaviour of silver clusters protected from atmospheric exposure, where the predominant ordered isomer is face-centred cubic. We propose that the formation of surface oxide or sulphide species resulting from air exposure can account for this deviation in stable isomer. This interpretation is consistent with density functional theory calculations based on silver nanoclusters, in the size range 147-201 atoms, on which methanethiol molecules are adsorbed. An understanding of the effects of ambient exposure on the atomic structure and therefore functional properties of nanoparticles is highly relevant to their real-world performance and applications.

## Abstract (web summary)

The atomic structures of metallic nanoparticles dictate their chemical and physical properties, making their structural study under different chemical environments of importance. Here, electron microscopy finds that silver nanoparticles primarily adopt an icosahedron structure under air exposure, while previous reports showed that the face-centred cubic isomer is favoured when the nanoparticles are kept in vacuum.

## Introduction

The structural evolution of metallic nanoparticles (NPs) under different chemical environments^1^ plays a pivotal role in their chemical and physical properties^2^. Understanding, and preferably controlling, metallic nanoparticles at the atomic scale in real-world conditions is a prerequisite to tuning their properties, especially for catalysis. Nanometre-sized atomic clusters of silver (Ag) and gold (Au) are the subjects of intensive research interest as model systems. In the case of Ag, there is no clear consensus on the preferred atomic structures and the influence of environmental parameters^3–6^. The appearance of icosahedral (Ih) structures in materials known as face-centred-cubic (FCC) in bulk is of a longstanding interest in the literature^7^, and still remains of high-interest nowadays^8^. A recent report showed that when Ag clusters are synthesised in a vacuum, protected in inert gas and characterised in a vacuum with only the briefest ambient exposure^5,6^, decahedral (Dh) and especially FCC isomers dominate the ordered fraction (50-80%) of clusters, with Ih structures almost completely excluded^5,6^. The classical view from atomistic modelling^9^ is that the Ih structure is competitive for small magical numbers while Dh dominates over the range 1–10000 atoms^10^, but the theoretical models for Ag NPs traditionally take no account of environmental effects, except when thiolates are considered as synthesis ligands^11^. The crossover sizes between the diverse symmetries are expected to be dependent on the material, chemical environment, synthesis method and support, among other factors. In particular, when working with highly reactive metal like silver toward oxygen and/or sulphur leading to surface species, the determination of the most favourable isomers become even more challenging, as such species are strongly bound^12,13^. However, these surface species will not bias the identification of the crystallinity of the nanoclusters as long as silver nanoparticles keep their structure and symmetry in their core. Moreover, the isomer distribution may reflect kinetic factors, such as trapping in structures set by the early-stage growth of the clusters^14,15^. Here, we combine statistical analysis, to determine the distribution of structural isomers of Ag clusters grown by the synthesis in a vacuum and then exposed to air at length, with density functional theory (DFT) calculations considering the effect of methanethiol adsorption on the stability of three isomers. We demonstrate a clear tendency of the Ag clusters to deviate from FCC to Ih isomers over a broad size range (up to 1000 atoms). This trend differs from a recent study of Ag clusters stored in ultrahigh vacuum (UHV) conditions that show the absence of Ih isomers over this size range (below 5%). The preferential adsorption of methanethiol shell on Ih isomer over other symmetries is predicted from DFT models of Ag clusters exposed to air, in the range of 147-201 atoms, in agreement with our measurements. Ih clusters better accommodate such contamination thanks to a larger adsorption site density.

## Results

### Silver nanoparticles preparation

The Ag clusters samples were produced with a Matrix Assembly Cluster Source (MACS)^16,17^ and deposited on amorphous carbon supports for transmission electron microscopy experiments (TEM). This new type of nanoparticle beam source has been developed for the scale-up production of nanoparticles up to the gram scale per hour. In contrast to the standard cluster beam source, which produces a large proportion of charged particles, the MACS produces mostly neutral charge particles hindering the use of mass filtration. Traditional cluster beam source consists of condensing hot atoms in cold gas, while the MACS implies the impact of an energetic ion beam (Argon in that case) upon a condensed matrix of rare gas atoms filled with atoms of desired material (Ag, for instance). The impact of an ion induces a cascade of multiple collisions inside the cold matrix that initiates cluster nucleation until the ion beam impact releases it from the matrix. Other details of the experimental conditions can be found in the methods section. The nanoclusters were soft-landed directly onto the support in high-vacuum conditions. We expect a degree of aggregation once the clusters land on the support, depending on the availability of local defect sites to prevent cluster diffusion^18,19^. The degree of coalescence is not pertinent to the conclusions drawn. The samples were subsequently exposed to an ambient atmosphere for an extended period of time (several weeks) before atomic-resolution imaging was performed with a JEOL ARM200F STEM at Diamond Light Source, operating at 200 keV. The microscope was equipped with a Cs corrector and high-angle annular dark field detector (HAADF).

### Statistical analysis of Ag isomers proportions

**Fig. 1:**
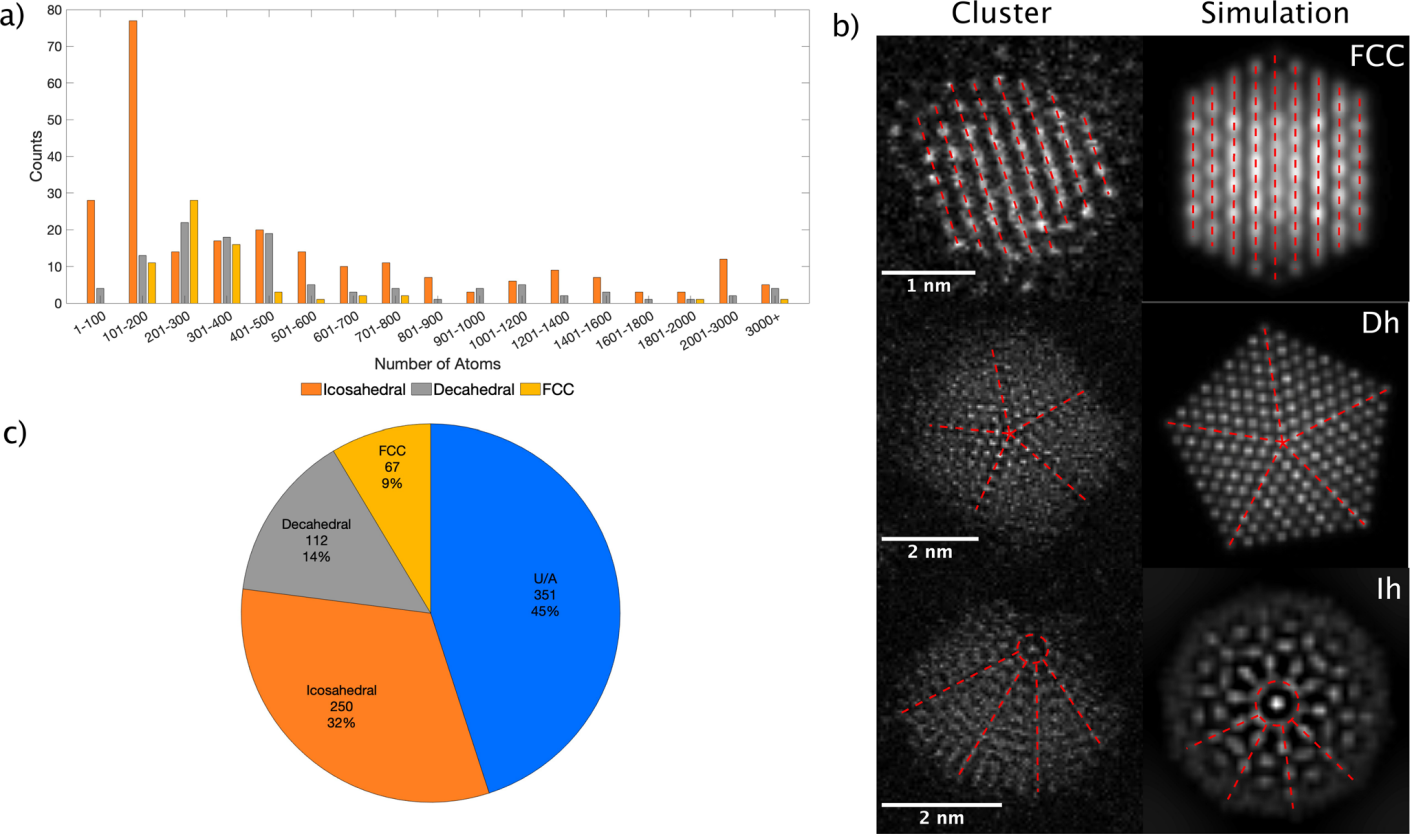
Size distribution and statistical analysis of the isomer proportions for Silver nanoparticles exposed to air on amorphous carbon. **a** Number of clusters as a function of size in the range of 1–3000 atoms, split into the different isomer structures observed. **b** HAADF-STEM images of three different morphologies and their corresponding assignments using the Simulation Atlas method; Face-centred cubic (FCC), decahedral (Dh), and icosahedral (Ih) with 309, 1233, and 835 atoms, respectively. Dotted red lines and red circles area as a guide for the eye. **c** Statistics for silver cluster symmetries averaged over the whole size range of (**a**). The U/A assignments correspond to unidentified or amorphous (glassy) structures.

The cluster size distribution was obtained from the HAADF images by integrating the HAADF intensity of the whole clusters and carefully normalising it against the single Ag atom intensity^20^. As seen in Fig. 1a, a range of cluster sizes was observed (1–3000 atoms), and the large majority (90%) lies in the range of 1–1000 atoms. A minority of large clusters was identified and we expect this fraction to be dominated by cluster coalescence on the surface. The specific isomer symmetry of each individual cluster was obtained by the Simulation Atlas method^20^, wherein each experimental image was compared with simulated images of numerous orientations of each of the main competing structural symmetries^21,22^. Three different ordered isomers have been identified, icosahedral, decahedral, and face-centred cubic nanoparticles, as illustrated in Fig. 1b. These structures co-exist with glassy (amorphous) cluster structures. Amongst the ordered isomers, Ih is the most common structure (32%), followed by Dh (14%), and finally, FCC (9%), as shown in Fig. 1c.

## Discussion

These results are broadly in line with a previous experimental study for silver clusters, in which Ih symmetry was identified in a size range lower than 5 nm^23^. However, as is the historical norm, the effect of ambient exposure was not discussed^23^. As noted above, the storage of the Ag cluster in inert gas^6^ resulted in predominantly FCC and Dh structures with few Ih clusters at a size of 309 ± 7 atoms. Note that the set of U/A clusters combines the clusters that cannot be identified using the Simulation Atlas method, e.g., amorphous (glassy) nanoclusters, with a small number of clusters presenting poor signal to noise.

The main challenge before us is to explain the predominance of the Ih isomer amongst the order Ag clusters in this study, by contrast with the very different isomer distribution observed in previous studies^5,6^, in which the clusters were stored in inert gas and Ih is a tiny minority structure. In fact, two lines or arguments converge to support an explanation of structural isomer modification due to air exposure, i.e., after cluster formation and deposition, a conclusion which should inform the interpretation/reinterpretation of other experimental studies of Ag nanoparticles. The first draws on an experimental study of chemically-synthesised, thiolated AgAu alloy nanoparticles^24^. Inspection of the experimental data, also from aberration-corrected HAADF-STEM, shows that in the limit of pure or almost pure Ag clusters, Ih is very much a dominant ordered isomer. For thiolated (Au_x_Ag_1-x_)_312±55_ clusters containing more than 80% Ag, the proportion of Ih is itself more than 80%. This result strongly suggests the idea that exposure of naked Ag clusters to sulphur-containing molecules in the atmosphere (the means by which silverware contaminates) may stabilise the Ih motif.

### First-principles adsorption thermodynamics for air contaminated Ag clusters

**Fig. 2:**
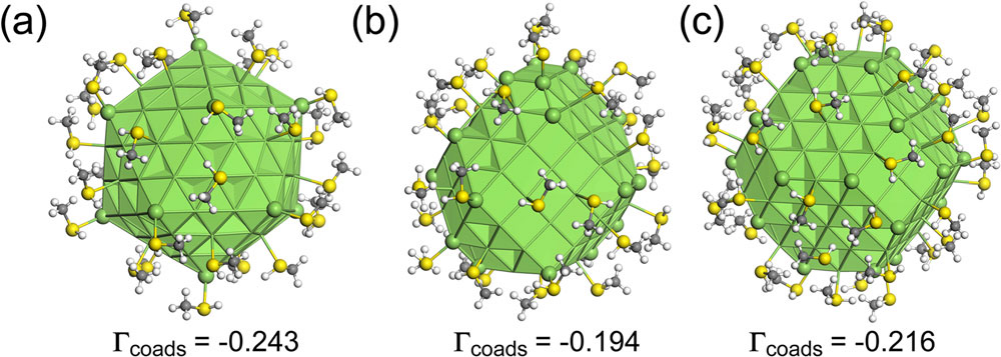
Illustration of silver nanoparticles covered by a shell of chemisorbed methanethiol molecules (model contaminants) at similar coverages on different structures, according to density functional theory (DFT) optimisations. **a** Regular icosahedron Ag147ico (Icosahedral symmetry, diameter Ø = 1.66 nm, θ = 0.35 ML, 32 contaminant molecules), **b** Strongly irregular truncated octahedron Ag147sito (Face-centred cubic, C1 symmetry, Ø = 1.70 nm, θ = 0.33 ML, 32 contaminant molecules) and **c** Regular truncated octahedron Ag201rto (Face-centred cubic, Oh symmetry, Ø = 1.80 nm, θ = 0.36 ML, 44 contaminants). Adsorption energies Γ_coads_, are expressed in J m^−2^. The colour labels for atoms are silver in green, sulphur in yellow, carbon in grey and hydrogen in white. In the silver nanoparticles, the vertices are represented by balls, whereas the bonds between silver atoms belonging to facets and edges are depicted by joint sticks. ML means monolayer.

The second argument for the influence of the chemical environment on the stability of small silver clusters comes from density functional theory calculations (DFT), which we have developed to explore the effect of sulphur addition. Specifically, we used a dispersion-corrected PBE-D3 DFT functional (zero-damping formalism), as justified in our previous work^6^ to consider the adsorption of methanethiol on Ag clusters of three different morphologies (icosahedral Ag147ico and strongly irregular Ag147sito and regular Ag201rto truncated octahedral clusters), inside a cubic box of 125 nm^3^. Methanethiol, which is a neutral and moderately bound molecule on silver, was selected as a ligand model representative of sulphur-containing air contaminant. Based on a systematic study of isolated methanethiol adsorption, shells of this model sulphur-containing contaminant molecule were optimised on the clusters (see Fig. 2 for the most stable configurations and the number of contaminants). Thanks to the choice of various morphologies of Ag NP models (icosahedral, strongly irregular and regular truncated octahedral) and the consideration of various adsorption sites for methanethiol contaminants, the effect of change on their metallic coordination on adsorption properties could have thus been addressed. To make comparison possible, a similar surface coverage was chosen (θ ≈ 1/3 ML) for each cluster structure; corresponding to surface molecule densities (SMD) of 2.69, 2.04, and 2.24 molecules.nm^−2^ on Ag147ico, Ag147sito, and Ag201rto, respectively. Noteworthy is that the SMD on the Ag147ico is significantly larger than on the octahedral clusters, due to higher adsorption site density (7.7 sites.nm^−2^ for Ag147ico versus 6.1–6.2 sites.nm^−2^ for Ag147sito and Ag201rto). After geometry optimisations, the structures of the adlayers of contaminant molecules remained globally similar to their initial configurations. No desorption or physisorption was registered, meaning that the considered coverage is below saturation. The stability of the contaminant methanethiol shells on Ag clusters has been evaluated by calculating the total adsorption energy (co-adsorption energy in the sense of more than one molecule) Γ_coads_ referenced to the naked Ag cluster and gaseous methanethiol. This was normalised to the Ag NP surface area. Γ_coads_ is larger on the Ag147ico cluster (−0.243 J m^−2^) than on the octahedral structures (−0.194 and −0.216 J m^−2^ for Ag147sito and Ag201rto, respectively). The predicted larger exothermicity for adsorption on the icosahedral cluster (in line with the higher adsorption site density) is consistent with the experimental statistics, which show the pre-eminence of Ih forms over FCC clusters amongst the ordered structures observed. Hence, DFT results and experiments of chemically-synthesized thiolated Ag clusters are compatible with our observations after air exposure.

## Conclusion

The principal result of this work is that the exposure of Ag clusters (deposited on amorphous carbon films) to the air for a period of weeks leads to the predominance of the icosahedral isomer amongst the ordered structures observed, consistent with previous research (that did not consider the effect of the environment), but contrasting with the case where the clusters are preserved in an inert gas (in which case the Ih structure is barely evident). Thus, air exposure appears to switch the cluster structure: although Ag probably oxidises more rapidly than it reacts with sulphur, subsequent adsorption of sulphur-containing contaminant molecules stabilise the Ih isomer, consistent with our DFT model calculations and with the observed structure of chemically-synthesised Ag clusters capped with phenylethanethiolates molecules. More generally, the results expose the profound role of environmental exposure upon the energetics thus, properties of nanoparticles, an understanding that needs to influence both the reinterpretation of results published in the literature and the interpretation and design, of new experiments. It seems that, both at the fundamental level, and in terms of the real-world functional properties of nanomaterials, the atoms at the surface must be fully accounted for.

## Methods

The Ag clusters used in this work were prepared with a matrix assembly cluster source (MACS) system. First, a matrix is prepared on oxygen-free copper support mounted on a cold stage that is cooled to around 20 K by a closed-loop cryo-cooler (Sumitomo Heavy Industries, RDK-500E). The matrix is then created by depositing metal atoms of Ag onto the support from a thermal effusion cell (Createc, HTC), while simultaneously introducing Argon (Ar) gas into the chamber. Then, once generated, the matrix is sputtered with an Ar ion beam (Gencoa ion source) to generate the Ag clusters, which are soft-landed onto a carbon TEM grid.

The morphology imaging of the Ag clusters was performed by a JEOL ARM200F STEM at Diamond Light Source, operating at 200 keV. The microscope was equipped with a Cs corrector and a high-angle annular dark field detector (HAADF).

## Data Availability

The datasets generated during and/or analysed during the current study are available from the corresponding authors upon reasonable request.
